# On the role of surface morphology in impacting-freezing dynamics of supercooled droplets

**DOI:** 10.1038/s41598-024-61826-5

**Published:** 2024-06-01

**Authors:** S. R. Hosseini, M. Moghimi, N. M. Nouri

**Affiliations:** School of Mechanical Engineering, Iran University of Science and Technology, Tehran, 13114-16846 Iran

**Keywords:** Numerical simulation, Supercooled droplet, Surface morphology, Rebound and adhesion, Freezing process, Mechanical engineering, Physics

## Abstract

A thorough understanding of droplet impact and freezing is vital in preventing ice accretion on many outdoor devices. This simulation-based study investigated the effect of surface morphology on the impacting-freezing process of a supercooled droplet. Also, the variations of Weber number and supercooling temperature were studied numerically. The droplet impact and freezing process were simulated with the volume of fluid method and freezing model. A more accurate simulation was achieved by modeling the supercooled droplet and the dynamic contact angle. At the given ranges of the input parameters, the main factors that guaranteed droplet rebounding after collision were determined. The supercooling temperature and the groove width should be above 266 K and less than 0.21 mm, respectively. The droplet should also maintain its cohesion and integrity during impact. Creating grooves on a surface is novel and paves a new way to understand the impact and solidification of water droplets in supercooled conditions.

## Introduction

Depending on its purity, water can remain liquid below freezing temperature, called supercooled water. Water in this state, called a metastable state, may freeze with the slightest disturbance. Freezing of supercooled droplets on different surfaces has destructive environmental effects^1^. For example, the accumulation of ice on the wings and other forward-facing components of aircraft or unmanned aerial vehicles (UAVs) may change their lift and drag forces or even cause them to fall^2,3^. More generally, this phenomenon can be challenging for most outdoor devices like power transmission lines and pin insulators^4–6^, ships and boats, atmospheric applications and clouds, traffic lights, windshields of vehicles, heat exchangers, the food industry, and many other subjects. Therefore, it is necessary to have a complete understanding of this phenomenon and ways to prevent it.

Using superhydrophobic surfaces was one of the first suggestions to prevent the droplet from freezing after impacting the surface. However, many superhydrophobic surfaces investigated do not work successfully at temperatures below zero^7,8^. Hence, these surfaces cannot always be used in general icing conditions. One reason is that the properties of the fluid in solidification are strongly temperature-dependent. Another reason is the metastable state of supercooled water and the special issues related to it, which make this phenomenon complex^9^.

Many researchers have investigated the main parameters of impacting-freezing supercooled droplets. Some of these parameters are thermal, such as surface temperature^10,11^, supercooling temperature^12^, and temperature dependence of fluid properties, especially during solidification^9^. The second category is the surface and fluid parameters, such as surface wettability^13,14^, surface elasticity^15^, dynamic contact angle (DCA)^16^, Weber number^17^, droplet splashing^18–20^, spreading factor—which is described as the ratio of spreading or wetted diameter to the initial diameter ($\beta_{max} = D_{max} /D_{0}$)—^21,22^, and the evolution of the droplet shape^23^. Some other investigated parameters are related to the phase change process, such as freezing onset^24^, solidification time^25^, freezing propagation^26^, supercooled droplet and solidification modeling^27^, and ice adhesion strength^28^.

For example, Wang et al.^16^ used DCA to improve the simulation accuracy. They showed that at low Weber numbers, the non-dimensional maximum spreading factors of the room temperature and supercooled water droplet were nearly identical but were quite different at high Weber numbers. In addition, they demonstrated the independence of the stable spreading factor from the Weber number, with the former increasing with a contact angle and a supercooling temperature decrease. Kong et al.^24^ concluded that the frozen part of the droplet after spreading does not depend on the surface wettability at temperatures under 258.15 K. In comparison, at temperatures higher than 263.15 K, the mean frozen spreading ratio depends only on wettability. A linear transition region exists in this ratio amid these two temperature points. According to Sun et al.^21^, at temperatures higher than 264.15 K, the freezing spreading rate becomes almost independent of the supercooling temperature yet positively correlates with surface wettability. Below this temperature, surface wettability is less influential, and the freezing spreading rate rises with supercooling.

Zhang et al.^10^ prepared a diagrammatic representation of droplet bouncing and freezing in correlation with Weber number, contact angle, and supercooling temperature, with generic margins for complete rebound and sticking. To make the result more applicable to in-flight icing, Zhou et al.^29^ investigated the behavior of multiple droplets simultaneously in icing conditions. Also Shinan et al.^17^ and Tretola et al.^30^ investigated the high-velocity impact of millimeter-sized droplets. Yao et al.^31^ examined impacting-freezing on extremely cold surfaces with temperatures from 173.15 to 233.15 K, emphasizing how the surface temperature affects the freezing front speed and total solidification time. Accordingly, there was a slight drop in the final contact area with a sharp drop in surface temperature below 233.15 K. Zhang et al.^32^ examined this phenomenon on similar extremely cold surfaces and obtained the effects of surface temperature on the spreading factor and freezing morphologies. Yao et al.^11^ focused on the effects of ultra-cold surfaces on the rebound/adhesion of the droplet and established a regime map for three different behaviors of droplet rebounding.

Wang et al.^33^ examined how surface temperature, impact velocity, and inclined angle affect the dynamics of impacting droplets, revealing that the temperature limit of fully rebound moves to a greater surface temperature when the impact velocity increases. Nonetheless, the impact velocity did not significantly affect the spreading time and contact time. The finest temperature limit of complete rebound was 238.15 K, with a Weber number of 19 and an inclined angle of 30°, representing a considerable improvement relative to a horizontal surface's temperature limit. Another investigation was made by Liu et al.^34^ describing the effects of concave and covex surfaces on the impacting-freezing process.

Attarzadeh and Dolatabadi^35^ simulated the icephobic behavior of superhydrophobic coatings. Pillars with square cross-sections were used to model the superhydrophobic topology. They examined the effects of surface topology and thermal characteristics on maximum spreading factor, droplet penetration, freezing inception, and solidification time. However, it seems that the dimensions used in their simulations, such as the height of the pillars, the distance between pillars, and the droplet diameter, were too small and in the micrometer range, causing various limitations. In addition, the contact time of the droplet with the surface was a few microseconds and was too short to be visible. The reason is their intended aeronautical application; in fact, the diameter of the droplets in the clouds is a few micrometers or even smaller. They actually intended to model a superhydrophobic surface roughness physically, which is entirely different from changing the morphology of a superhydrophobic surface and making grooves in it. Furthermore, if the surface morphology is such that the air beneath the droplet can escape from the sides during impact (e.g., arrays with squared cross-sectional pillars), an air cushion beneath the droplet would not form because there is no air compression.

To the best of the authors’ knowledge, although many researchers have simulated the impact and freezing of a droplet, most have investigated the impact and freezing separately, have not considered the supercooled droplet effects, or have not used a grooved surface. In this study, the impact and freezing characteristics of a millimeter-sized water droplet were simulated under supercooled conditions. For the first time, surfaces with different groove sizes were used with multiple Weber numbers and different supercooled temperatures. This technique can significantly reduce the droplet contact area to prevent droplet freezing. Also, the surface morphology was considered in such a way that after the droplet impacts the surface, the air under the droplet becomes trapped, condenses to some extent, and provides no escape route, meaning that it can be considered an air cushion.

## Methodology

### Governing equations

To model the process of droplet collision, a multiphase flow model must be selected. Thus, the Volume of Fluid (VOF) approach^36^ can be utilized in the transport equations of each phase. To capture the interface between the phases, a continuity equation was solved for the volume fraction of each phase. The volume fraction definition is:

1 $$\gamma_{i} = \frac{{{\text{volume}}\,{\text{of}}\,i{\text{th}}\,{\text{phase}}}}{{{\text{cell}}\,{\text{volume}}}},\quad i = 1, 2$$

where

2 $$\gamma_{1} + \gamma_{2} = 1$$

Here, *γ*_1_ denotes the volume fraction of air, while *γ*_2_ denotes the liquid phase volume fraction consisting of unfrozen water–ice and frozen ice. Hence, the air volume fraction value in each cell is as follows:

3 $$n\gamma_{1} = \left\{ {\begin{array}{*{20}l} 1 \hfill & {\quad if{\mkern 1mu}\,the{\mkern 1mu}\,cell{\mkern 1mu}\,contains{\mkern 1mu}\,air{\mkern 1mu}\,only} \hfill \\ {0 < \gamma_{1} < 1} \hfill & {\quad if{\mkern 1mu}\,the{\mkern 1mu}\,cell{\mkern 1mu}\,contains{\mkern 1mu}\,both{\mkern 1mu}\,air{\mkern 1mu}\,and{\mkern 1mu}\,liquid} \hfill \\ 0 \hfill & {\quad if{\mkern 1mu}\,the{\mkern 1mu}\,cell{\mkern 1mu}\,contains{\mkern 1mu}\,liquid{\mkern 1mu}\,only} \hfill \\ \end{array} } \right.$$

To describe the conservation of mass for each phase, Eq. (4) can be used:

4 $$\frac{\partial }{\partial t}\left( {\gamma_{i} \rho_{i} } \right) + {\mathbf{\nabla }} \cdot \left( {\gamma_{i} \rho_{i} {\mathbf{u}}} \right) = 0,\quad i = 1, 2$$

However, a single momentum equation is solved in all cells in the domain:

5 $$\frac{\partial }{\partial t}\left( {\rho {\varvec{u}}} \right) + {\mathbf{\nabla }} \cdot \left( {\rho {\varvec{uu}}} \right) = - {\mathbf{\nabla }}p + {\mathbf{\nabla }} \cdot \mu \left[ {\nabla {\varvec{u}} + \left( {\nabla {\mathbf{u}}} \right)^{{\text{T}}} } \right] + \rho {\varvec{g}} + {\varvec{F}}_{frz} + {\varvec{F}}_{Tens}$$

where ***F***_*frz*_ (which is introduced in section "Freezing model") denotes the freezing process effects and ***F***_*Tens*_ denotes the surface tension effects. The surface tension force was calculated using the continuum surface force (CSF) model^37^. This model agrees well with experimental findings in the literature^38^. It expresses ***F***_*Tens*_ as:

6 $${\varvec{F}}_{Tens} = \sigma \frac{{\rho \kappa {\mathbf{\nabla }}\gamma_{1} }}{{\overline{\rho }}}$$

where $\overline{\rho }$ is the average density of two phases and *κ* refers to the interface's curvature. This parameter is determined as follows:

7 $$\kappa = {\mathbf{\nabla }} \cdot {\varvec{n}},\quad {\varvec{n}} = \frac{{{\mathbf{\nabla }}\gamma_{1} }}{{\left| {{\mathbf{\nabla }}\gamma_{1} } \right|}}$$

in which ***n*** is the normal unit vector of the phase interface. Fluid properties like density (*ρ*), thermal conductivity (*k*), and dynamic viscosity (*μ*) vary with temperature in each phase. They are described as:

8 $$\rho = \mathop \sum \limits_{i} \gamma_{i} \rho_{i} ,\quad k = \mathop \sum \limits_{i} \gamma_{i} k_{i} ,\quad \mu = \mathop \sum \limits_{i} \gamma_{i} \mu_{i} \quad i = 1, 2$$

All the basic physical properties values used in our simulation were obtained from handbooks^39^. When a droplet impacts a surface, the tangential line to the droplet (at the contact line) makes an angle with the horizontal surface, called the contact angle (*θ*). The contact angle typically varies with contact line velocity, so it is referred to as the DCA. To determine the DCA, the empirical model described by Kistler^40^ was adopted, which provides results that are in good agreement with experimental data^41^. DCA is described as:

9 $$DCA = f_{Hoff} \left[ {Ca + f_{Hoff}^{ - 1} \left( {\theta_{{{\text{eq}}}} } \right)} \right]$$

where $f_{Hoff} \left( x \right)$ and $f_{Hoff}^{ - 1} \left( x \right)$ are the Hoffman function and its inverse^42^, obtained from:

10 $$f_{Hoff} = arccos\left\{ {1 - 2tanh\left[ {5.16\left( {\frac{x}{{1 + 1.31x^{0.99} }}} \right)^{0.706} } \right]} \right\}$$

in which $Ca = \mu V_{CL} /\sigma$ is the capillary number, *V*_*CL*_ is the velocity at the contact line, and *θ*_*eq*_ is the equilibrium contact angle, which is described as:

11 $$\theta_{eq} = \left\{ {\begin{array}{*{20}l} {\theta_{{adv,\quad V_{CL} > 0}} } \hfill \\ {\theta_{{rec,\quad V_{CL} < 0}} } \hfill \\ \end{array} } \right.$$

while *θ*_*adv*_ is the advancing and *θ*_*rec*_ is the receding contact angle.

### Method of supercooling temperature consideration

Conventionally, in the impacting process of a supercooled water droplet on a cold solid surface, like what happens at room temperature droplets and surfaces, the spreading, retreating, oscillating, and stable stages are observed^43^. By including the cold surface and supercooled conditions, external stages like supercooling, nucleation, recalescence, and freezing will be observed^44–46^. The impacting process causes vibration, so the nucleation process occurs once the droplet touches the surface^47^, and the recalescence stage begins and finishes in a fraction of a second. Therefore, ignoring the time of nucleation and recalescence, the new initial conditions at the beginning of the simulation (the impact moment) should be considered.

In other words, before impact, the supercooled droplet is completely liquid, and the velocity, diameter, and supercooling temperature of the droplet are *U*_0_, *D*_0_, and *T*_0_, respectively. Upon impact, the supercooled droplet rapidly converts to a mixture of water and ice. Its temperature returns to the solidification point *T*_*S*_ (273.15 K), and thus, its diameter and other characteristics change^48^. Hence, new initial conditions develop for starting the simulation, identified with the initial subscript. However, because of the comparison that may be made between supercooled conditions and normal conditions, the Weber number is still defined as:

12 $$We = \rho U_{0}^{2} D_{0} /\sigma$$

If an energy balance is established between the supercooling condition and the initial condition, the initial mass fraction of ice (*α*_*initial*_) at the mixture of water and ice is described as:

13 $$\alpha_{initial} = \frac{{c_{{water, T_{S} }} \left( {T_{S} - T_{0} } \right) }}{L}$$

The physical characteristics, like the latent heat of freezing, the density, specific heat, thermal conductivity, and diameter, can be described as:

14 $$L_{initial} = \left( {1 - \alpha_{initial} } \right)L$$

15 $$\rho_{initial} = \left( {1 - \alpha_{initial} } \right)\rho_{water} + \alpha_{initial} \rho_{ice}$$

16 $$c_{initial} = \left( {1 - \alpha_{initial} } \right)c_{water} + \alpha_{initial} c_{ice}$$

17 $$k_{initial} = \left( {1 - \alpha_{initial} } \right)k_{water} + \alpha_{initial} k_{ice}$$

18 $$D_{initial} = \left( {\frac{{\rho_{water} }}{{\rho_{initial} }}} \right)^{1/3} D_{0}$$

Generally, the ice fraction can be calculated by:

19 $$\alpha = \left\{ {\begin{array}{*{20}l} 1 \hfill & {\quad T < T_{Solidus} } \hfill \\ {\frac{{T_{Liquidus} - T}}{{T_{Liquidus} - T_{Solidus} }}} \hfill & {\quad T_{Solidus} < T < T_{Liquidus} } \hfill \\ 0 \hfill & {\quad T > T_{Liquidus} } \hfill \\ \end{array} } \right.$$

In Eq. (19), the liquidus/solidus temperature is the temperature at which the droplet is completely water/ice. In this work, the values of these temperatures are $T_{Liquidus} = 273.25 {\text{K}}$ and $T_{Solidus} = 273.05 {\text{K}}$^47^. So the initial temperature of the droplet will be:

20 $$T_{initial} = \left( {1 - \alpha_{initial} } \right)T_{Liquidus} + \alpha_{initial} T_{Solidus}$$

### Freezing model

As the VOF method is used to calculate mass and momentum equations, a suitable and accurate freezing model should be used for the solidification calculations in the impacting process. As stated in Sec. "Governing equations", the initial mixture of water and ice is considered a liquid phase in this study. Therefore, its latent heat and other thermodynamic properties are obtained from Eqs. (14)–(20). In modeling the freezing process, the porosity-enthalpy approach was used to calculate the liquid fraction in each cell. Enthalpy balance must be maintained in all cells, whether filled with liquid or solid. The total enthalpy consists of the sum of latent heat and sensible enthalpy.

21 $$h = h_{Sens} + h_{Lat}$$

The sensible enthalpy in Eq. (21) is calculated as follows:

22 $$h_{sens} = h_{Ref} + \mathop \smallint \limits_{{T_{Ref} }}^{T} cdT$$

while *h*_*Ref*_ is the reference enthalpy. Latent heat is:

23 $$h_{Lat} = \left( {1 - \alpha } \right)L_{initial}$$

Hence, in our solidification model, the energy equation for all phases can be:

24 $$\frac{\partial }{\partial t}\left( {\rho h} \right) + {\mathbf{\nabla }} \cdot \left( {\rho {\varvec{u}}h} \right) = {\mathbf{\nabla }} \cdot \left( {k\user2{\nabla }T} \right)$$

When the enthalpy-porosity model is used, a mushy zone that works like a porous medium is also proposed. In very pure water, there is no mushy zone. However, in most simulations, it should be considered. In each cell, the porosity equals the liquid fraction and reduces from 1 to 0 upon freezing the water droplet. When the droplet freezes completely in each cell, the porosity becomes zero, and the velocity should be zero too. Thus, the freezing term (***F***_*frz*_) must be added to the momentum equation to force the velocity to zero. The appropriate form is:

25 $${\varvec{F}}_{frz} = \frac{{\alpha^{2} }}{{\left( {1 - \alpha } \right)^{3} + \varepsilon }}A_{mush} {\varvec{u}}$$

To prevent the denominator from becoming zero, a small number, *ε* = 0.001 should be added. The mushy zone constant, A_mush_, adjusts the rate at which the cell's velocity reaches zero. The larger it is, the more strongly the velocity moves toward zero within solidification. In the ANSYS Fluent user guide^49^, the recommended range for A_mush_ is from 10^4^ to 10^7^. If a smaller value is used, the obvious velocity can be seen in the solidified region. Larger values may cause oscillation in our simulation. The mushy zone also has a physical meaning related to freezing time. On the other hand, the supercooling temperature, *T*_0_, has a similar meaning. Thus, a relationship can be established empirically between these two variables. Table 1 presents this relation.

**Table 1:** Mushy zone constant values for different supercooling temperatures^10^.

Supercooling temperature *T*_0_ (K)	268.15	263.15	258.15	253.15
*A*_*mush*_	10^4^	5 × 10^4^	10^5^	5 × 10^5^

### Numerical simulation procedure

The two-phase flow in the domain could be assumed as two-dimensional axisymmetric. A square domain with a 5D_0_ length was considered, a no-slip wall boundary condition was chosen for the bottom surface, and a pressure outlet boundary condition was selected for the limits of the domain. A structured mesh was used to improve the efficiency of calculations and the accuracy of liquid–gas interfaces. The grid size was refined to 10 × 10 μm^2^ around the coordinate axis where the droplet flows. ANSYS Fluent, as computational software, was used to solve the equations. The fluid was incompressible, the solver was pressure-based, and the Kistler DCA model was employed by embedding a user-defined function (UDF) file. The time step was variable, controlled between 10 ^−9^ ~ 10 ^−6^ s by setting 0.05 for the maximum Courant number.

### Validation of numerical model

**Figure 1:**
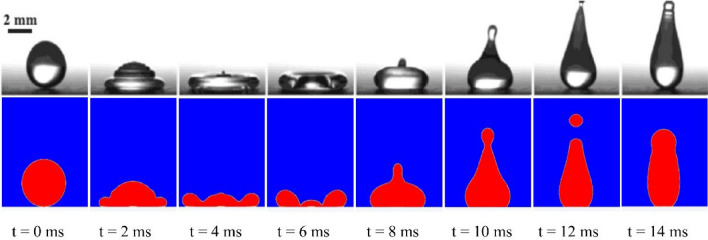
Comparison of the isothermal impact of a liquid droplet and surface between the present numerical results and the experimental observations by Zhang et al.^10^. Air is shown in blue, and water droplets in red.

**Figure 2:**
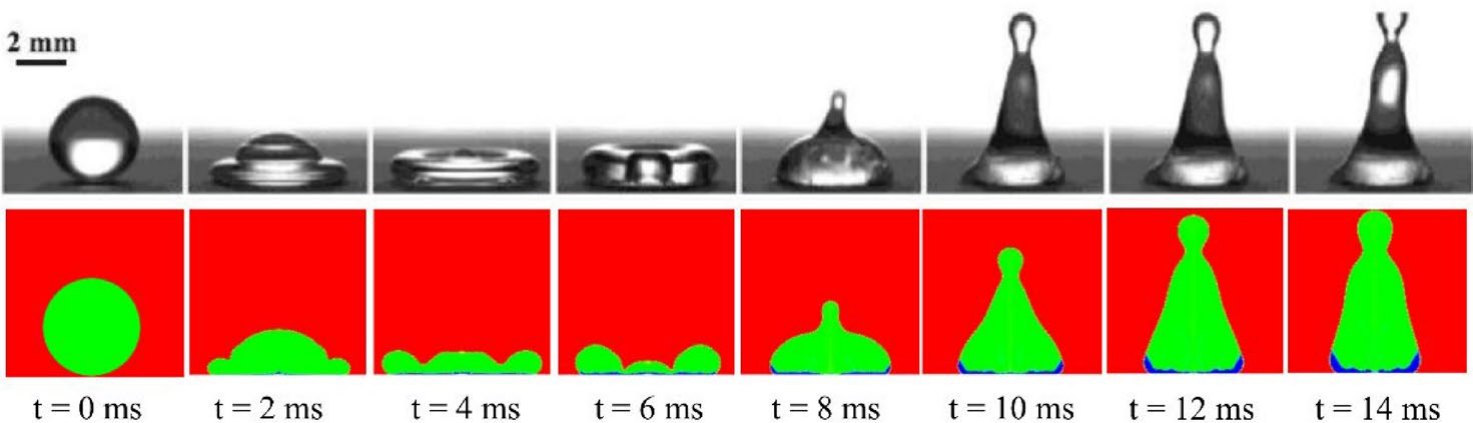
Comparison of the supercooled droplet impact on the cold surface between the present numerical results and the experimental observations by Zhang et al.^10^. Air is shown in red, a liquid water droplet in green, and ice in blue.

Before exploring the effect of surface morphology, to check the accuracy and reliability of our numerical results, they were compared with two experimental results of Zhang et al.^10^. In the first one, the isothermal impact of a droplet on a surface at ambient temperature was studied, and in the second one, the impact and freezing of a droplet on a surface under supercooled conditions were investigated. In both cases, the impact velocity was 0.7 m/s, the Weber number was 19.18, and the initial diameter of the droplet before impact was 2.84 mm. Both surfaces' advancing/equilibrium/receding contact angles were 162°/160°/158°. In the first case, the surface, air, and droplet temperatures were 288.15 K. In the second case, the surface temperature was 243.15 K, but the air and droplet temperatures were 268.15 K. As shown in Figs. 1 and 2, despite the slight difference between our numerical simulation and experimental observations, this deviation is admissible because of the presence of complexities at fluid flow and phase change in the simulation of a supercooled droplet like initial conditions and the empirical terms like mushy zone constant. As we see, the droplet rebounds from the surface in the first case. However, in the second case, the droplet attaches to the surface.

In the second case, the droplet is supercooled, which is the main reason behind its adhesion to the surface. The freezing process starts immediately after the impact. As the lower part of the droplet freezes, the spreading diameter becomes fixed. While the upper part of the droplet is still in a liquid state and tries to bounce off the surface, the droplet stretches, and its tip may even separate from the droplet and move up. In fact, the advancement of heat transfer and fluid flow are simultaneously observed, and heat transfer is coupled with fluid flow.

## Investigated cases

**Figure 3:**
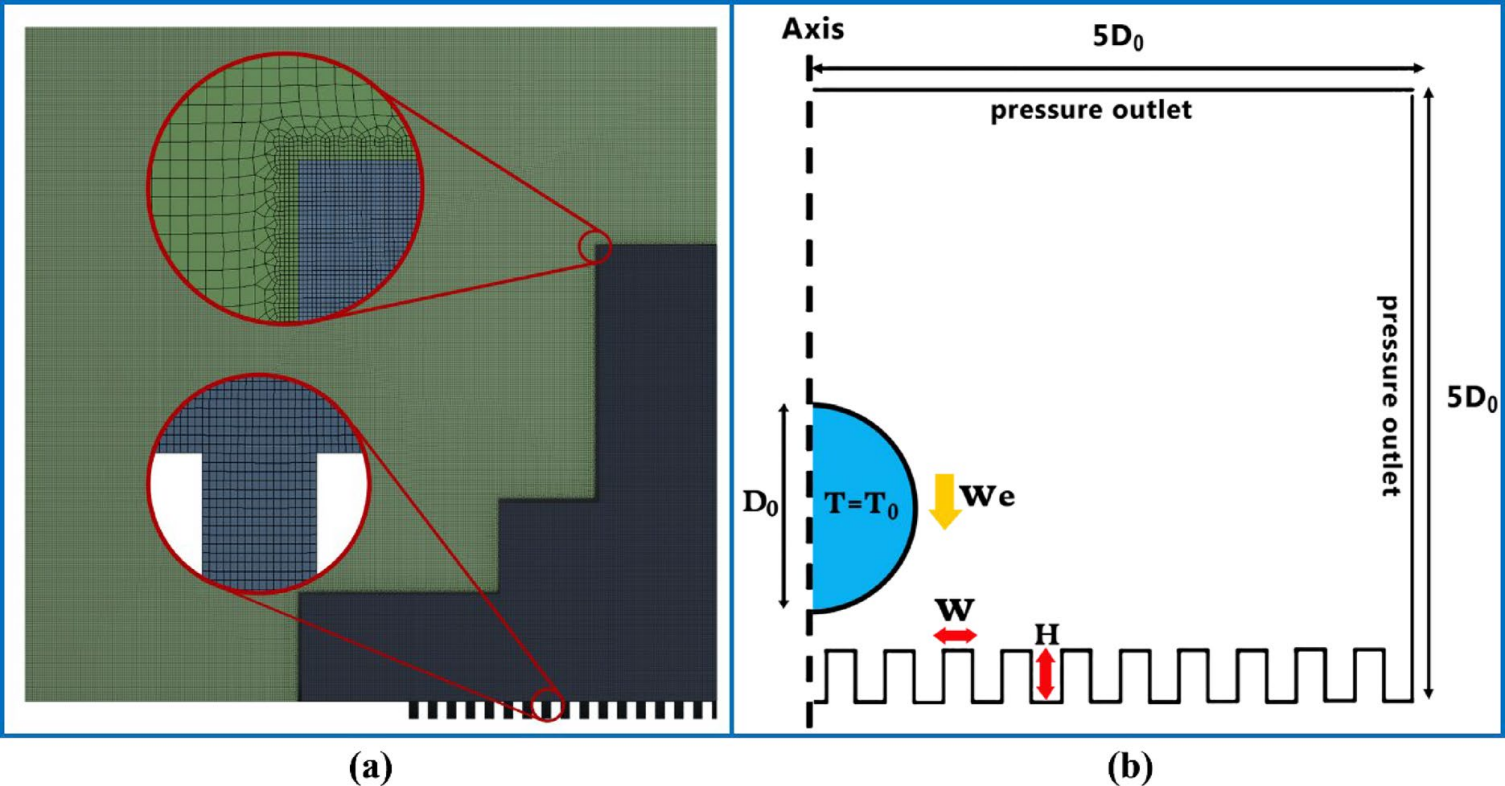
(**a**) Refined structured mesh and (**b**) schematic model of the initial and boundary conditions.

As mentioned in the previous sections, when the supercooled droplet impacts a flat and cold surface, it starts freezing immediately. Now, if some regular grooves are formed on the surface, the droplet may experience another situation because the significant reduction of the contact area may help it to rebound. The creation of air cushions under the droplet can also be another barrier against freezing at the surface. Note that creating a groove on the surface is entirely different from the issue of roughness because the surface roughness scales are micro and nano, while the width and height of the grooves are tens and hundreds of times the roughness scales. In the numerical analysis (Fig. 3), two geometrical parameters (groove dimensions, W and H), one fluid parameter (Weber number), and one heat transfer parameter (supercooled temperature, T_0_) were selected as the four effective parameters in the bouncing of the droplet from the surface.

The main aim for choosing these different parameters was to conduct a comprehensive study. Most of the numerical simulation settings in this section are in accordance with validated simulations. The DCA of the surface was embedded with a UDF code. The residuals of mass, momentum, and energy are lower than 5*e* − 6 in each iteration. The width of grooves and tongues are the same in all the numerical simulations, such that the contact surface is 50% in all cases. The diameter of the droplet is considered constant, and the value of the Weber number changes with the change in droplet impact velocity. The range of the Weber number in this study is from 19 to 50. At very low Weber numbers, the droplet does not have enough kinetic energy to rebound from the surface, and in high Weber numbers, the droplet is divided into several pieces as soon as the droplet impacts the surface. It could be possible to increase the Weber number to 80, but due to the presence of wide grooves on the surface, the droplet may break into several pieces immediately after hitting the surface. That is why the maximum limit of 50 has been set.

The range of supercooling temperature was limited between 253.15 and 273.15 K. As we know, the lower the temperature of the supercooled droplet, the more likely it is to freeze before impacting the surface because the supercooled droplet in this condition is less stable and more sensitive to environmental disturbances and heterogeneous nucleation. The ranges of the groove size are equal to each other, from 0.071 to 0.364 mm. The range of geometrical parameters of the droplet is selected based on the size of the droplet diameter as a characteristic length, approximately from *D*/32 to *D*/8. The selection of the temperature range was also based on the freezing temperature of the droplet as a characteristic temperature. Therefore, it can be said that all considerations and selected ranges are based on dimensional analysis.

As previously mentioned, the problems were solved in a two-dimensional axisymmetric domain. The axis is shown in Fig. 3. The output parameters were:

1. The maximum spreading factor of the droplet.
2. Time of droplet rebound from the surface.
3. Integration or fragmentation of the droplet due to impact and rebound.

Considering that this simulation is complex and the input parameters have mutual effects on each other, changing one of the parameters and keeping the others constant will not necessarily lead to the correct conclusion. Naturally, our computational cost will be very high to simulate all possible states. Therefore, using the design of experiments (DOE) within the given ranges, the input parameters were selected randomly using ANSYS DesignXplorer software, and the results were analyzed. Among the types of DOE methods, the optimal space-filling (OSF) design, the advanced version of the Latin hypercube sampling (LHS) design, was chosen. The LHS method divides the given range into equal sections according to our sampling number and randomly selects only one sample from each section. OSF is essentially an LHS design optimized through several iterations, maximizing the distance between points to achieve a more uniform distribution across the design space. Using the OSF method, 20 design points were selected randomly. The results are provided in Table 2. It should be noted that there is no specific order in the rows, due to the randomness of the values in Table 2. However, all the values are within the ranges announced in this section.

**Table 2:** Random design points using the optimal space-filling (OSF) method.

No. of case	Pillar height (mm)	Pillar width (mm)	Supercooling temperature (K)	Weber number
1	0.26695	0.13645	262.65	19.9505
2	0.33945	0.25245	256.65	29.1965
3	0.07845	0.29595	264.65	38.4425
4	0.28145	0.07845	266.65	36.9015
5	0.17995	0.15095	271.65	27.6555
6	0.35395	0.19445	267.65	41.5245
7	0.23795	0.28145	272.65	43.0655
8	0.31045	0.17995	257.65	47.6885
9	0.12195	0.22345	253.65	33.8195
10	0.16545	0.10745	258.65	44.6065
11	0.22345	0.33945	255.65	39.9835
12	0.32495	0.23795	268.65	24.5735
13	0.13645	0.16545	269.65	46.1475
14	0.19445	0.26695	261.65	49.2295
15	0.09295	0.09295	263.65	32.2785
16	0.25245	0.12195	254.65	30.7375
17	0.29595	0.35395	265.65	35.3605
18	0.20895	0.32495	259.65	23.0325
19	0.10745	0.20895	260.65	21.4915
20	0.15095	0.31045	270.65	26.1145

**Figure 4:**
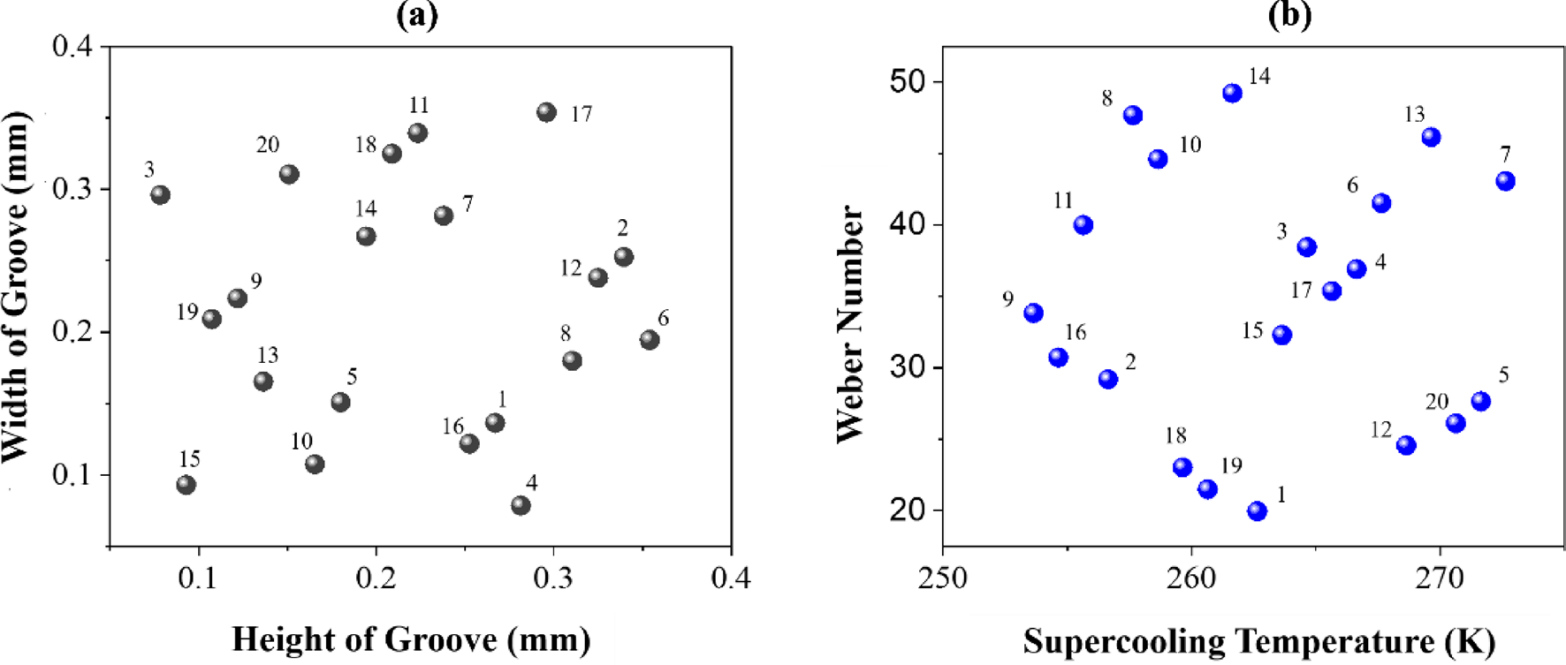
Distribution of (**a**) geometrical and (**b**) thermal-fluid input parameters in 20 simulations.

The distribution of input parameters is shown in Fig. 4.

As it is known, the OSF method covers almost the entire design space.

### Grid independence study

**Figure 5:**
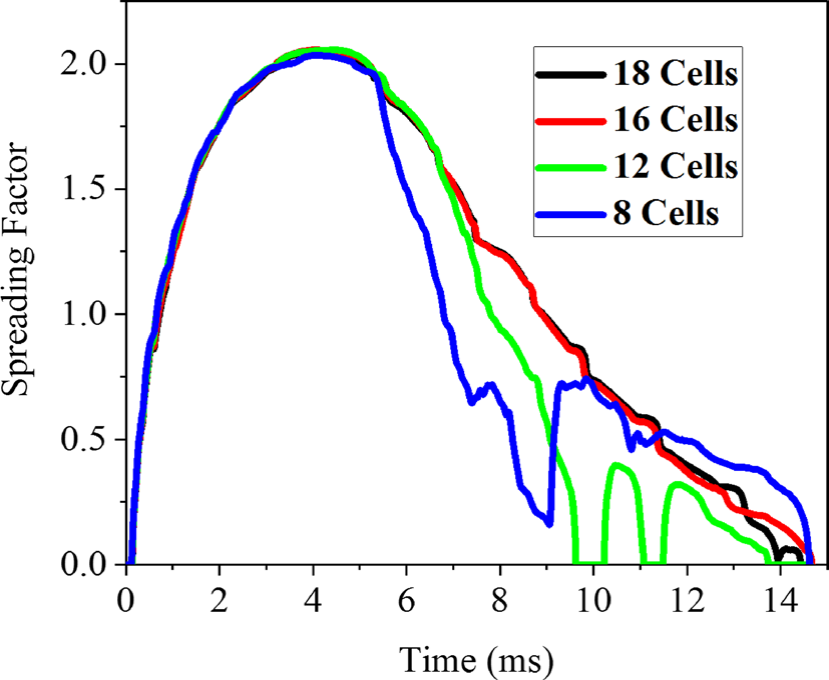
Grid independence test according to the number of cells in a groove width.

To achieve the most accurate and computationally efficient mesh, one of the cases in Table 2 (the 6th case) was selected as an example, and the spreading factor in 4 different grids was investigated. The results of these simulations are depicted in Fig. 5. The number of cells in a groove width increased continuously, and finally, according to Fig. 5, the grid with 16 cells in the groove width was selected as the best grid. The size of each cell in this chosen grid is 12 × 12 μm^2^. Therefore, in all the simulations of this study, the size of each grid cell would be the same, ensuring grid independence.

## Results and discussion

In all cases, due to the 50% reduction in the contact area of the droplet with the grooved surface, the probability of the droplet rebounding increased compared to a flat surface. However, this is not a general rule, as in some cases, the grooves increased the contact of the droplet with the surface, and the so-called trapping of the droplet lowered the possibility of droplet rebounding. In 13 of the 20 performed simulations, the droplet could rebound from the surface after impact, meaning that the probability of rebounding was 65%. The following results were obtained from the numerical simulation of these 20 cases:

### Effects of input parameters on the maximum spreading factor

**Figure 6:**
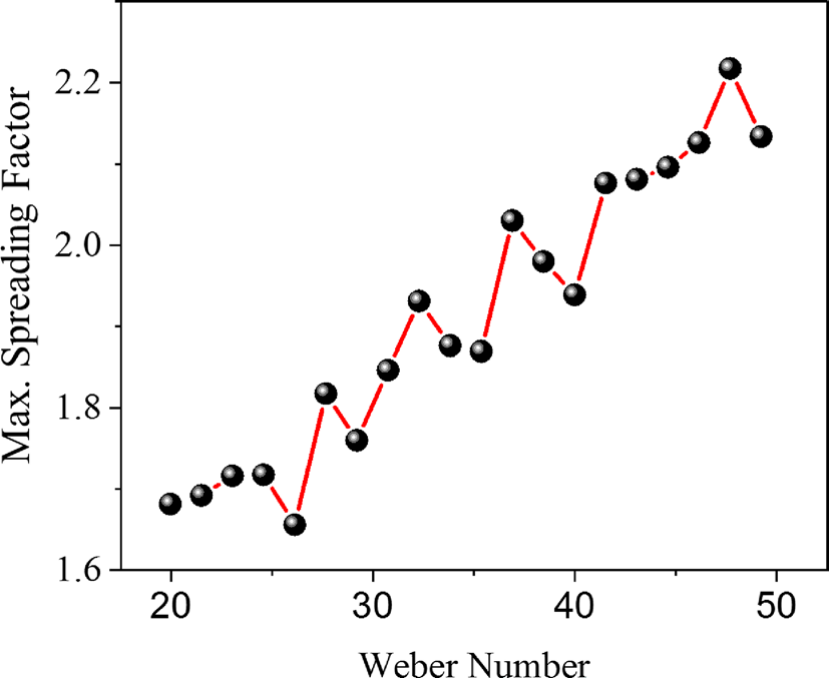
Weber number vs. maximum spreading factor for all 20 cases.

Due to the coupling of input parameters and their simultaneous change in each numerical simulation, simple and regular charts were not expected. The first chart shows the Weber number change according to the maximum spreading factor. As seen in Fig. 6, the general trend of the graph is ascending, which shows that one of the decisive parameters affecting the droplet maximum spreading factor is the Weber number.

In some cases, the chart's upward trend in Fig. 6 turned downward. One of the main reasons is that when the Weber number increased, the rest of the input parameters were not constant, and these changes caused a slight decrease in the maximum spreading factor of one design point compared to the previous.

**Figure 7:**
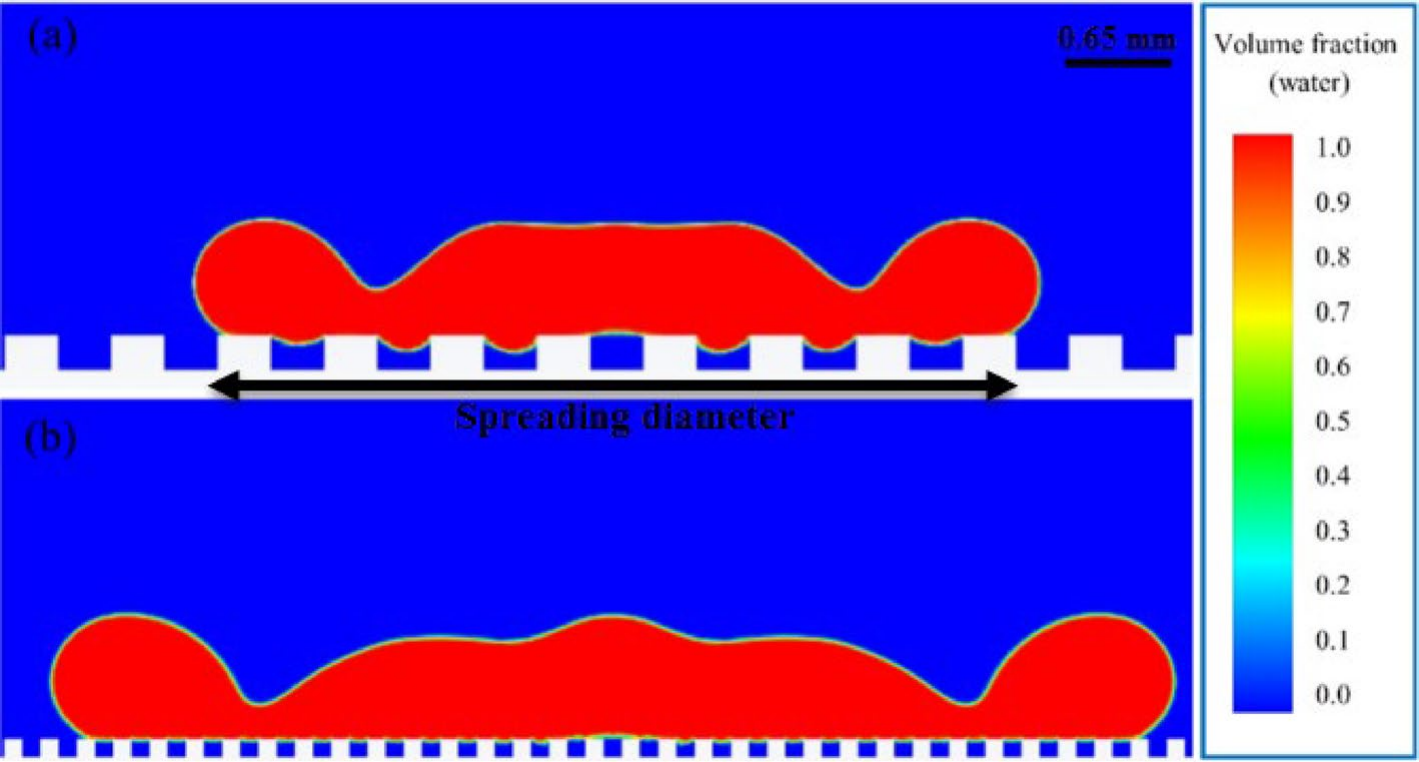
Effect of groove width on spreading: volume fraction contour at t = 3.9 ms in (**a**) the 18th case and (**b**) the 15th case. Air is shown in blue, and water droplets in red.

The large width of the grooves can also be another factor in reducing the maximum spreading factor. When the droplet reaches near maximum spreading, due to the reduction of horizontal momentum force, surface tension is the only force that keeps the droplet from falling inside the groove. If the groove width is relatively large, for example, in the 18th case (Fig. 7a), the gravity force in the grooves, just like a brake, prevents the droplet from spreading. However, if the surface is composed of narrower grooves, for example, in the 15th case (Fig. 7b), the effect of gravity will be much less, and the droplet spreads easily in the horizontal direction.

Droplet penetration inside the grooves proceeds until the surface tension and gravity balance each other. Because fluid changes are faster than thermal changes in this simulation, no remarkable influence of the supercooling temperature was observed.

### Effects of input parameters on integrity and rebounding time of droplet

**Figure 8:**
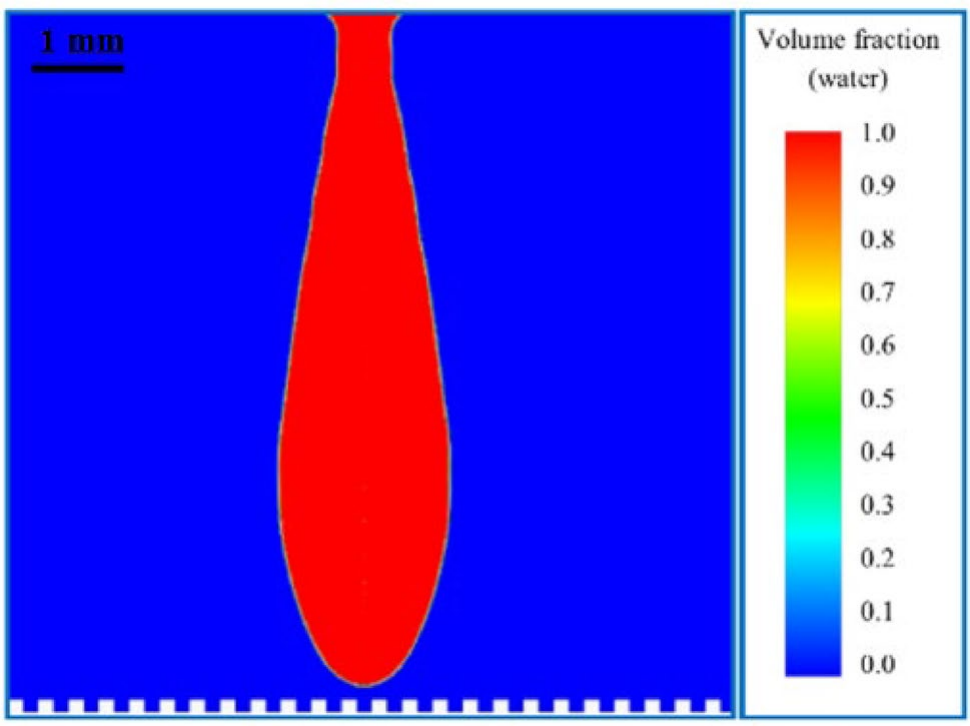
Separation of the droplet from the surface after impact: volume fraction contour at t = 15.7 ms in the 13th case. Air is shown in blue, and water droplets in red.

When a droplet impacts the surface, the spreading stage starts. After the droplet reaches its maximum spreading, it enters the receding stage. This stage consists of two parts. The first part is the movement of the droplet in the horizontal direction towards the axis (horizontal receding), and the second part is the effort of the droplet in the vertical direction (vertical receding) to rebound from the surface. The droplet momentum in a horizontal and vertical receding, depending on the value of the input parameters, may cause the droplet to fragment in the first or second part. This issue entirely affects the rebounding/adhering of the droplet from/to the surface. Among the 20 numerical simulations performed, in four cases, i.e., the 1st, 4th, 5th, and 13th cases, the droplet maintained its continuity and integrity both in the horizontal and vertical receding and easily rebounded from the surface. Therefore, it can be said that maintaining the integrity of the droplet is an essential factor in the separation of the droplet from the surface. In the 13th case (Fig. 8), the separation of the droplet from the surface was observed after impacting:

Because the Weber number has changed from 19 to 46 in these four cases, it can be said that whether or not the droplet is divided has nothing to do with the Weber number of the droplet. This matter is also true for the groove height because it also experiences significant changes.

The lowest supercooling temperature of the droplet in these four numerical simulations was 262.65 K. Hence, reducing the temperature to this value is not enough for the droplet to break into pieces or adhere to the surface. Although naturally, the reduction of supercooling temperature increases the probability of the droplet adhesion or fragmenting into several pieces. The maximum spreading factor of the droplet in these four cases varies widely. Although it seems the excessive spreading of the droplet and the thinning of its thickness is a key factor in the fragmentation, the numerical results do not show this. At least, it can be said that the increase in the spreading factor alone is not an effective factor in adhesion or disrupting the continuity of the droplet.

**Figure 9:**
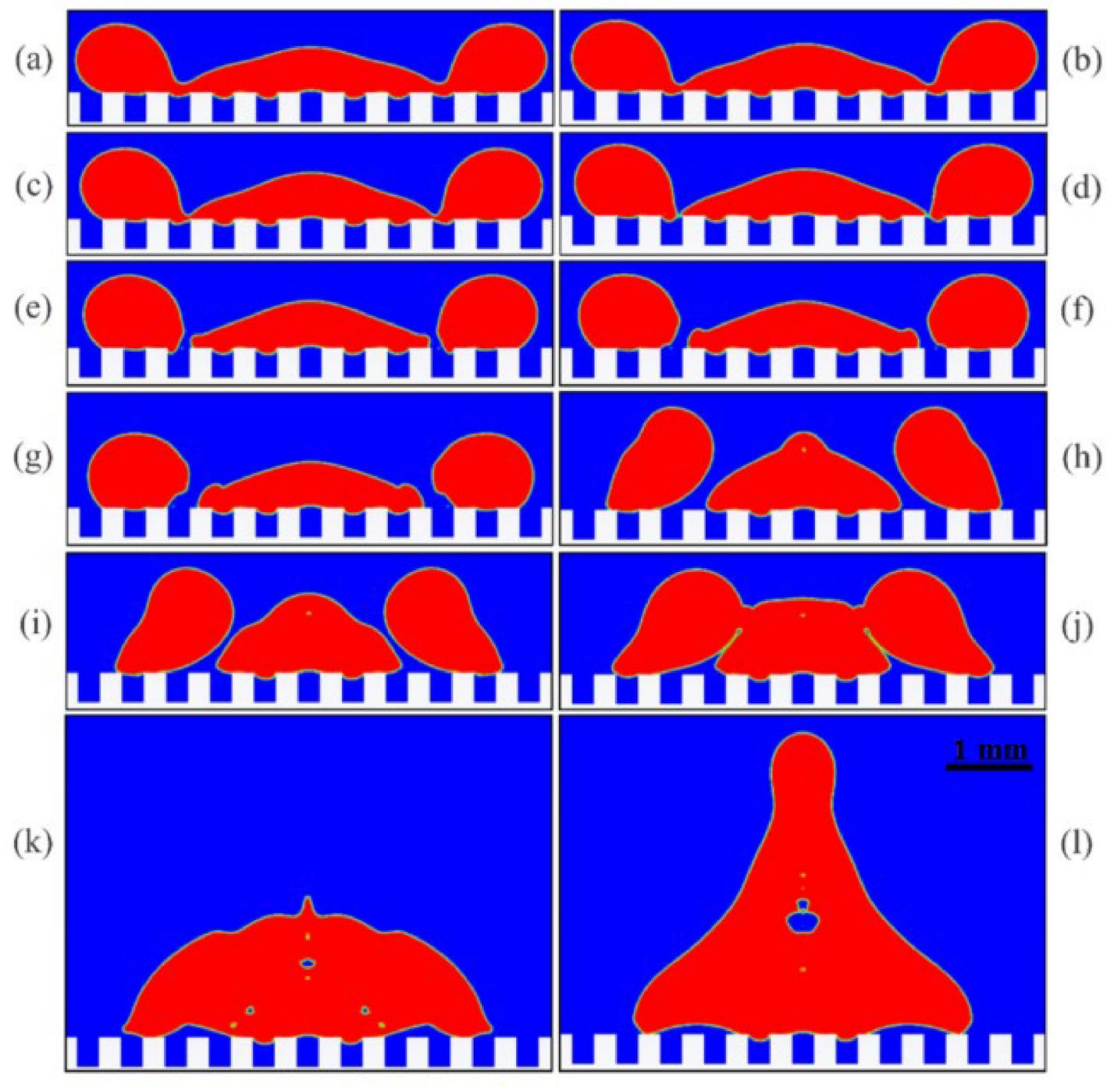
A large groove width causes the droplet to fragment into pieces: volume fraction contours at different times from the 2nd case, (**a**): t = 5.1 ms, (**b**): t = 5.3 ms, (**c**): t = 5.4 ms, (**d**): t = 5.5 ms (the moment of fragmentation), (**e**): t = 5.6 ms, (**f**): t = 5.7 ms, (**g**): t = 5.8 ms, (**h**): t = 7.2 ms, (**i**): t = 7.6 ms, (**j**): t = 8.0 ms (the moment of remerge), (**k**): t = 8.6 ms, (**l**): t = 10.6 ms. Air is shown in blue color, and water droplets in red color.

The groove width is the most important factor that seems to cause the droplet to fragment into pieces. The maximum groove width in these four cases was 0.165 mm, which is a safe value. However, other parameters would also have to be considered for larger values. This issue was predictable from the beginning. Because in a horizontal receding, when the droplet is moving towards the center, if the groove width is relatively large (like in Fig. 9 from the 2nd case), the droplet momentum helps gravity and overcomes the surface tension in the thinned part above the groove and finally divides the droplet into pieces.

When the droplet is divided into several parts, its kinetic energy is also divided, and a part of its kinetic energy is wasted in this process. Hence, even though the droplet pieces remerge (see Fig. 9), the merged droplet probably does not have enough energy to separate from the surface. However, in some cases, the droplet was finally able to separate from the surface despite being broken into several pieces. Therefore, breaking the droplet into several pieces increases the probability but does not necessarily cause the droplet to adhere to the surface.

**Figure 10:**
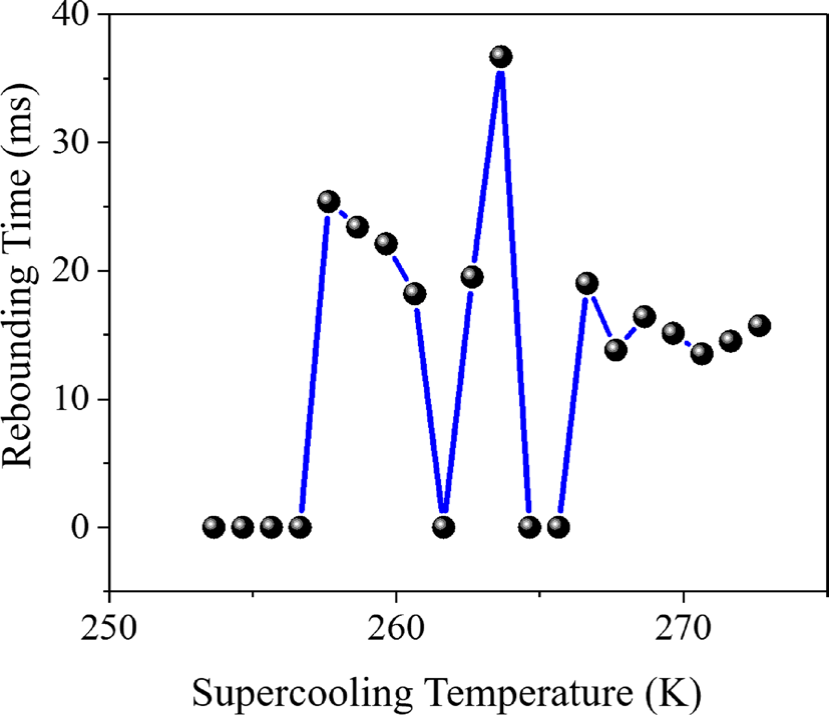
Supercooling temperature (K) versus rebounding time (ms): all 20 cases.

The effect of supercooling temperature can be seen in the rebounding of the droplet from the surface because enough time has passed for the heat transfer effects to occur. According to Fig. 10, it seems that the temperature of supercooling is one of the main factors of rebounding/sticking of the droplet from/to the surface. Because in all seven numerical simulations, where the supercooling temperature of the droplet was above 266 K (the 4th, 5th, 6th, 7th, 12th, 13th, and 20th cases), even though the other input parameters of the simulation varied widely, the droplet was able to bounce from the surface. On the other hand, in all four numerical simulations where the supercooled temperature of the droplet was less than 257 K (2nd, 9th, 11th, and 16th cases), despite extensive changes in other parameters, the droplet could not rebound from the surface and stuck to the surface. At temperatures between 257 and 266 K, in some cases, the droplet stuck to the surface; in others, the droplet could separate from the surface. This finding shows that the supercooling temperature, in this range, is not the dominant and decisive parameter in the droplet separation. All of these items can be seen in Fig. 10. Zero separation time in Fig. 10 means that the droplet cannot separate from the surface.

**Figure 11:**
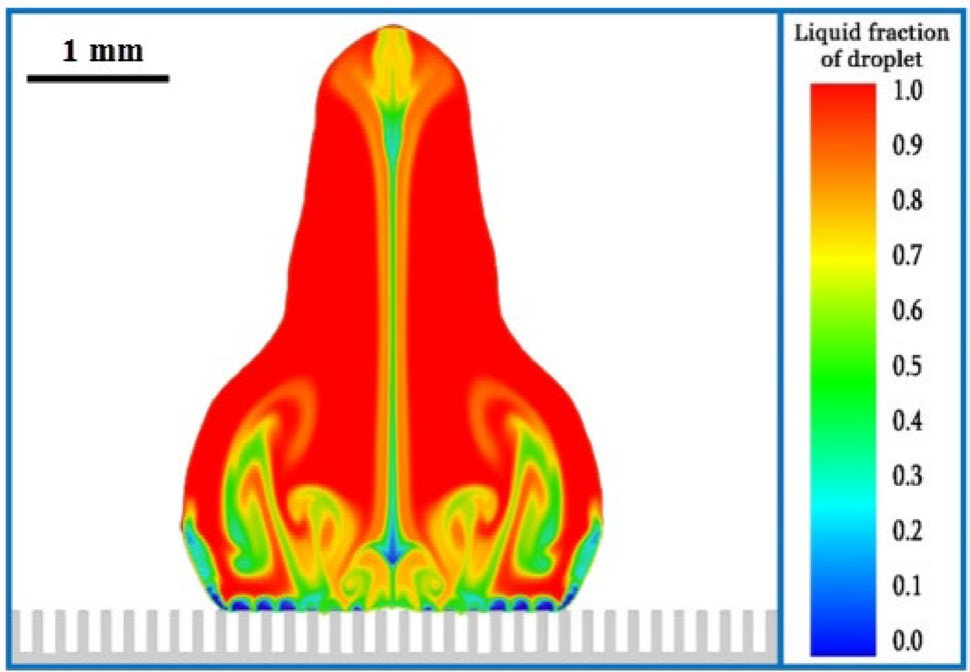
Grooves prevent the small frozen areas of the droplet from being continuous: phase change contour at 10 ms in the 4th case.

In examining the effect of the groove width on droplet separation, it can be said that a narrow groove width is an important factor in the ability of the droplet to separate from the surface. Because out of ten numerical simulations where the groove width was less than 0.21 mm, in nine of them, the droplet could separate from the surface (1st, 4th, 5th, 6th, 8th, 10th, 13th, 15th, and 19th cases). The droplet could not bounce from the surface only in one numerical simulation (16th case) due to the extremely low supercooling temperature (254.65 K), as explained in the previous section. In these nine cases, the Weber number and the groove height varied widely but did not affect the separation issue. Reducing the contact surface to 50% is the most important advantage of narrow groove width. Also, the grooves create a distance between the frozen areas in a successive manner and prevent the formation of a continuous and integrated frozen region. In Fig. 11, from the 4th case, these issues are visible.

A further explanation is that in some cases (e.g., Fig. 11), the fragmented contact regions freeze. However, when the droplet bounces from the surface, due to the dynamics that are formed inside the droplet and the significant kinetic energy of the droplet, small frozen parts separate from the surface and cannot prevent bouncing. These fragmented freezing regions cause the droplet to face multiple small local resistances that it can more easily overcome to bounce relative to a single large source of resistance.

**Figure 12:**
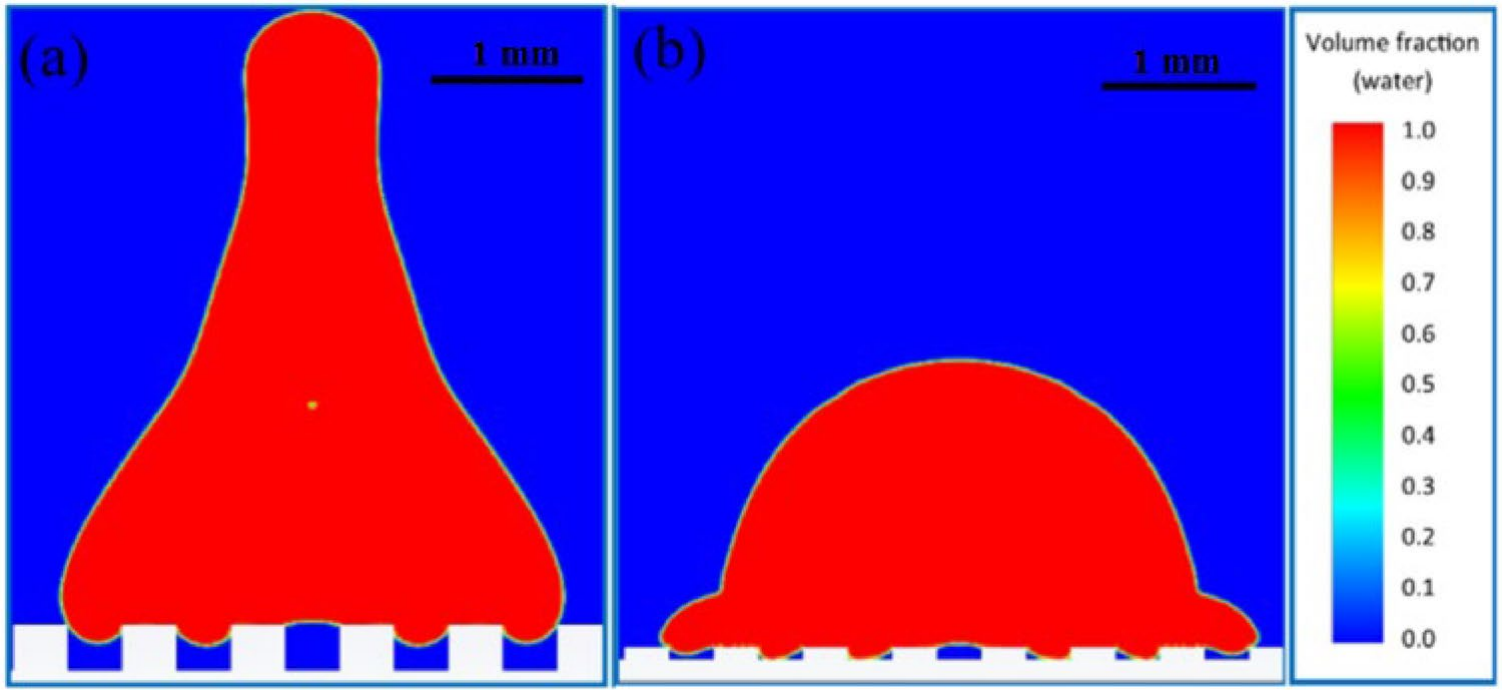
(**a**) The droplet was trapped within the grooves: volume fraction contour at t = 9.5 ms in the 17th case. (**b**) Contact of the droplet with the bottom of the grooves: volume fraction contour at t = 1.4 ms in the 3rd case. Air is shown in blue, and water droplets in red.

Regarding the effect of the groove height on the separation of the droplet, no remarkable effect was seen in the numerical simulations. The only thing that can be stated is that if, in addition to the groove height, the groove width is also relatively large (e.g., Fig. 12a from the 17th case), the droplet gets trapped inside these grooves and cannot separate from the surface. That is, in some conditions, it has a negative effect on the separation of the droplet from the surface.

Among 20 numerical simulations, the lowest groove height value was in the 3rd case. An interesting phenomenon here is the impact of the droplet on the bottom of the grooves (Fig. 12b), which happened in none of the 19 other numerical simulations. The first reason for this issue is the relatively high speed of the droplet, and the second (which seems to be a more important factor) is the relatively large groove width along with a low groove height.

**Figure 13:**
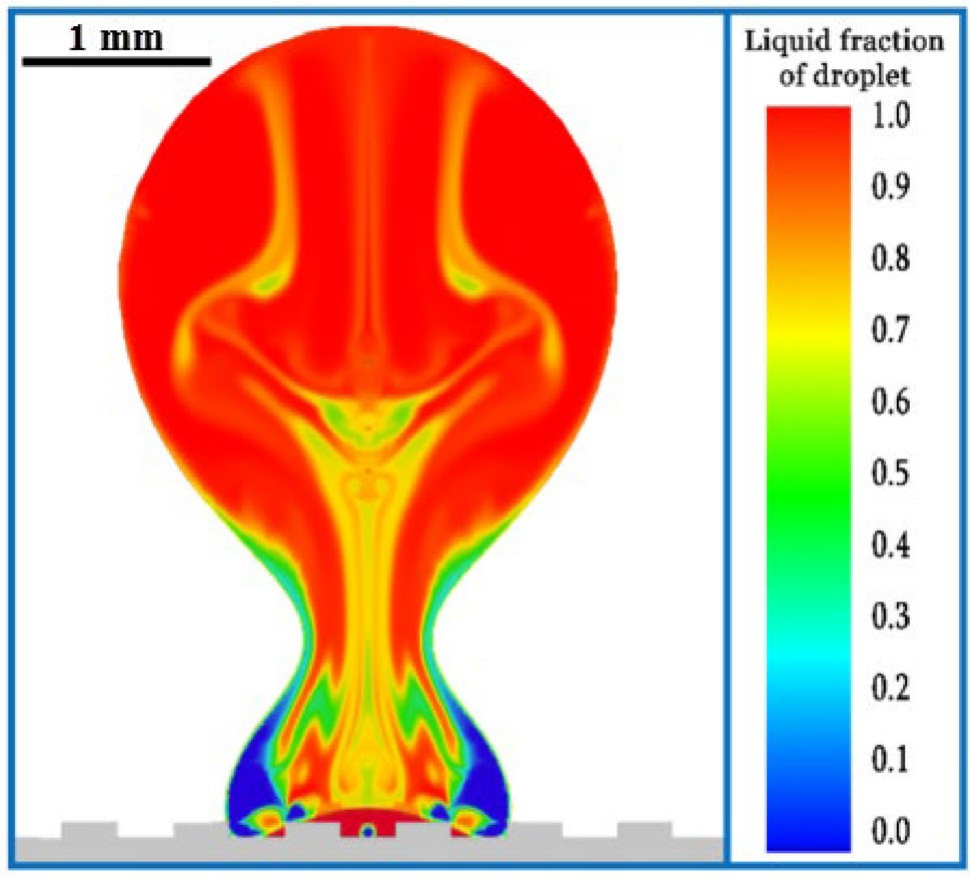
Freezing and adhering the droplet to the surface: phase change contour at t = 28.1 ms in the 3rd case.

In the 3rd simulation, the surface tension force did not need to resist the gravity force. Rather, the solid surface at the bottom of the grooves caused the droplet to find close support, and after the space inside the groove was filled, it continued to spread. Therefore, first of all, the contact surface of the droplet increased and was no longer 50%; it was even more than 100% compared with a flat surface, which thermally increased the probability of the droplet sticking to the surface. Secondly, because the droplet completely filled the space between the grooves, it got trapped inside the grooves and could not be separated from the surface. The simulation results presented in Fig. 13 fully confirm this analysis.

Although in terms of supercooling temperature values, the 3rd simulation was at an average level, the geometric factor (groove) helped the thermal condition and saved time for it to easily freeze a significant volume of the droplet near the surface. Hence, the droplet stuck between the grooves, making it impossible to rebound. As seen in Fig. 13, although a significant part of the droplet was trying to separate from the surface, the surface adhesion was so strong that all the kinetic energy of the droplet was lost in this thermal-fluidic struggle. Finally, the droplet rested on the surface. There were similar contours in the 9th, 11th, and 16th cases, where, in some, the kinetic energy of the droplet's upper part overcame the continuity force of the droplet, and the droplet was divided into two parts while rebounding from the surface.

## Conclusions

The main goal of this study was to investigate the effect of surface morphology (the width and height of the grooves) on the collision of a supercooled droplet with a cold surface. Also, the effects of Weber number and supercooling temperature on this collision were investigated. For this purpose, using DOE (the OSF method), random values for the above four parameters were selected for 20 numerical simulations. Some of the main results of these 20 simulations were as follows:

1. Due to the 50% reduction in the contact area of all cases, the probability of the droplet rebounding from the surface increased, so it rebounded from the surface in 13 out of 20 cases. In all cases where the droplet could maintain its continuity and cohesion, it separated from the surface. Fragmentation of the droplet into several pieces did not necessarily cause it to stick to the surface, but it increased the probability.
2. The most influential parameter on the maximum spreading factor was the Weber number, and the groove width was the most important factor that seemed to cause the droplet to fragment into pieces. The maximum safe value of the groove width was 0.165 mm.
3. The temperature of supercooling was one of the main factors of rebounding/sticking of the droplet from/to the surface, as all droplets with a temperature above 266 K bounced from the surface, and all with a temperature below 257 K adhered to the surface.
4. A narrow groove width facilitated the separation of the droplet from the surface: out of the ten cases where the groove width was below 0.21 mm, the droplet separated from the surface in nine cases. The most important advantage of a narrow groove width is that besides reducing the contact surface to 50%, it creates a distance between the frozen areas in a successive manner and prevents the formation of a continuous and integrated frozen region.
5. Regarding the effect of groove height on the separation of the droplet, except at the minimum value, no remarkable effect was seen in the numerical simulations.

## Letters

*h*: Enthalpy (J kg^−1^)
*A*_*mush*_: Mushy zone constant
*k*: Thermal conduction coefficient (W m^−1^ K^−1^)
*We*: Weber number
***u***: Velocity vector (m s^−1^)
*P*: Pressure (N m^−2^)
***n***: Unit normal vector (m)
*Ca*: Capillary number
*T*: Temperature (K)
*L*: Latent heat of solidification (kJ kg^−1^)
*C*: Specific heat (J kg^−1^ K^−1^)
*H*: Groove height (mm)
*W*: Groove width (mm)
*D*: Diameter (mm)

## Greek symbols

*γ*: Volume fraction
*ρ*: Density (kg m^−3^)
*μ*: Dynamic viscosity (kg m^−1^ s^−1^)
*κ*: Curvature (m^−1^)
*θ*: Contact angle
*σ*: Surface tension (N m^−1^)
*α*: Ice mass fraction
*β*: Spreading factor

## Subscripts

0: Before impact (time = 0)
*Ref*: Reference value
*S*: Solidification point
*i*: The ith phase
*Tens*: Surface tension
*Frz*: Freezing process
*Hoff*: Hoffman function
*CL*: Contact line
*Eq*: Equilibrium
*adv*: Advancing
*rec*: Receding
*Sens*: Sensible
*Lat*: Latent

## References

[1] Lv J, Song Y, Jiang L, Wang J (2014). Bio-inspired strategies for anti-icing. ACS Nano.

[2] Mosher, F., Schaum, D., Herbster, C., & Guinn, T. Analysis of causes of icing conditions which contributed to the crash of continental flight 3407. In *14th Conference on Aviation, Range, and Aerospace Meteorology* (2010). https://commons.erau.edu/db-applied-aviation/21/. Accessed Jan. 3, 2023.

[3] Deng H, Chang S, Song M (2020). Numerical investigation on the performance and anti-freezing design verification of atomization equipment in an icing cloud simulation system. J. Therm. Anal. Calorim..

[4] Han X, Jiang X (2019). Effect of DC electric field on water droplets’ movement and icing process on insulator. Cold Reg. Sci. Technol..

[5] Liao R (2015). Ice accretion on superhydrophobic insulators under freezing condition. Cold Reg. Sci. Technol..

[6] Naterer GF (2011). Multiphase transport processes of droplet impact and ice accretion on surfaces. Cold Reg. Sci. Technol..

[7] Dorrestijn M, Jung S, Megaridis CM, Raps D, Das A, Poulikakos D (2011). Are superhydrophobic surfaces best for icephobicity?. Langmuir.

[8] Hejazi V, Sobolev K, Nosonovsky M (2013). From superhydrophobicity to icephobicity: Forces and interaction analysis. Sci. Rep..

[9] Zhang X, Wu X, Min J, Liu X (2018). Supercooled water droplet impact on cold plates having different temperatures and contact angles. Int. Heat Transf. Conf..

[10] Zhang X, Liu X, Wu X, Min J (2020). Impacting-freezing dynamics of a supercooled water droplet on a cold surface: Rebound and adhesion. Int. J. Heat Mass Transf..

[11] Yao Y, Wu K, Yang R, Zhang H, Yang W, Li C (2024). Effects of surface temperature and Weber number on the dynamic and freezing behavior of impacting water droplets on a superhydrophobic ultra-cold surface. Appl. Therm. Eng..

[12] Zhang X, Liu X, Wu X-M, Min J-C (2020). Experimental and modeling research on the impact and freezing of a supercooled water droplet. J. Eng. Thermophys..

[13] Singh NS, Jitniyom T, Navarro-Cía M, Gao N (2024). Droplet impact on doubly re-entrant structures. Sci. Rep..

[14] Huang X, Gates I (2020). Apparent contact angle around the periphery of a liquid drop on roughened surfaces. Sci. Rep..

[15] Weisensee PB, Tian J, Miljkovic N, King WP (2016). Water droplet impact on elastic superhydrophobic surfaces. Sci. Rep..

[16] Wang Y, Wang Q, Ju L, Han D, Xue Y (2021). Numerical analysis on dynamics and thermodynamics of a supercooled water droplet considering the dynamic contact angle. Phys. Fluids.

[17] Shinan C, Liang D, Mengjie S, Mengyao L (2019). Numerical investigation on impingement dynamics and freezing performance of micrometer-sized water droplet on dry flat surface in supercooled environment. Int. J. Multiph. Flow.

[18] Wang Q (2021). Effects of surface roughness on splashing characteristics of large droplets with digital inline holographic imaging. Cold Reg. Sci. Technol..

[19] Quetzeri-Santiago MA, Castrejón-Pita JR, Castrejón-Pita AA (2021). Controlling droplet splashing and bouncing by dielectrowetting. Sci. Rep..

[20] Yonemoto Y, Tashiro K, Shimizu K, Kunugi T (2022). Predicting the splash of a droplet impinging on solid substrates. Sci. Rep..

[21] Sun M-M, Kong W-L, Wang F-X, Liu H (2019). Effect of surface wettability on the law of supercooled large droplet impact and heat transfer. K. Cheng Je Wu Li Hsueh Pao/J. Eng. Thermophys..

[22] Yonemoto Y, Kunugi T (2017). Analytical consideration of liquid droplet impingement on solid surfaces. Sci. Rep..

[23] Yao Y, Li C, Zhang H, Yang R (2017). Modelling the impact, spreading and freezing of a water droplet on horizontal and inclined superhydrophobic cooled surfaces. Appl. Surf. Sci..

[24] Kong W, Wang L, Bian P, Liu H (2022). Effect of surface wettability on impact-freezing of supercooled large water droplet. Exp. Therm. Fluid Sci..

[25] Lu M, Song M, Pang X, Dang C, Zhang L (2022). Modeling study on sessile water droplet during freezing with the consideration of gravity, supercooling, and volume expansion effects. Int. J. Multiph. Flow.

[26] Berberović E, Schremb M, Tuković Ž, Jakirlić S, Tropea C (2018). Computational modeling of freezing of supercooled water using phase-field front propagation with immersed points. Int. J. Multiph. Flow.

[27] Tembely M, Attarzadeh R, Dolatabadi A (2018). On the numerical modeling of supercooled micro-droplet impact and freezing on superhydrophobic surfaces. Int. J. Heat Mass Transf..

[28] Chen T, Cong Q, Sun C, Jin J, Choy KL (2018). Influence of substrate initial temperature on adhesion strength of ice on aluminum alloy. Cold Reg. Sci. Technol..

[29] Zhou X (2023). Bounce behaviors of double droplets simultaneously impact cold superhydrophobic surface. Int. J. Heat Mass Transf..

[30] Tretola G, Vogiatzaki K (2022). Unveiling the dynamics of ultra high velocity droplet impact on solid surfaces. Sci. Rep..

[31] Yao Y, Li C, Tao Z, Yang R, Zhang H (2018). Experimental and numerical study on the impact and freezing process of a water droplet on a cold surface. Appl. Therm. Eng..

[32] Zhang X, Li K, Zhu Z, Fang WZ, Zhu FQ, Yang C (2024). Droplet impact and freezing dynamics on ultra-cold surfaces: A scaling analysis of central-concave pattern. Appl. Therm. Eng..

[33] Wang X, Tang Z, Xu B, Chen Z (2021). Anti-freezing characteristics of water droplet impinging the superhydrophobic surface: An experimental and predictive study. Appl. Surf. Sci..

[34] Liu X, Guo Y, Min J, Zhang X, Wu X (2024). Impact and freezing coupling processes of supercooled water droplets on cold superhydrophobic spheres at low Weber numbers. Appl. Therm. Eng..

[35] Attarzadeh R, Dolatabadi A (2019). Icephobic performance of superhydrophobic coatings: A numerical analysis. Int. J. Heat Mass Transf..

[36] Hirt CW, Nichols BD (1981). Volume of fluid (VOF) method for the dynamics of free boundaries. J. Comput. Phys..

[37] Brackbill JU, Kothe DB, Zemach C (1992). A continuum method for modeling surface tension. J. Comput. Phys..

[38] Šikalo Š, Wilhelm H-D, Roisman IV, Jakirlić S, Tropea C (2005). Dynamic contact angle of spreading droplets: Experiments and simulations. Phys. Fluids.

[39] Haynes, W. M. *Handbook of Chemistry and Physics*, 95th ed (2014).

[40] Kistler SF (1993). Hydrodynamics of wetting. Wettability.

[41] Ji B, Song Q, Yao Q (2017). Numerical study of hydrophobic micron particle’s impaction on liquid surface. Phys. Fluids.

[42] Hoffman RL (1975). A study of the advancing interface. I. Interface shape in liquid—gas systems. J. Colloid Interface Sci..

[43] Khojasteh D, Kazerooni M, Salarian S, Kamali R (2016). Droplet impact on superhydrophobic surfaces: A review of recent developments. J. Ind. Eng. Chem..

[44] Wang H (2021). Bouncing behavior of a water droplet on a super-hydrophobic surface near freezing temperatures. Int. J. Heat Mass Transf..

[45] Li FF, Liu J (2010). Thermal infrared mapping of the freezing phase change activity of micro liquid droplet. J. Therm. Anal. Calorim..

[46] Nitsch K (2009). Thermal analysis study on water freezing and supercooling. J. Therm. Anal. Calorim..

[47] Zhang X, Liu X, Wu X, Min J (2018). Simulation and experiment on supercooled sessile water droplet freezing with special attention to supercooling and volume expansion effects. Int. J. Heat Mass Transf..

[48] Blake J, Thompson D, Raps D, Strobl T (2015). Simulating the freezing of supercooled water droplets impacting a cooled substrate. AIAA J..

[49] A. Inc. *ANSYS Fluent User Guide* (ANSYS Inc, 2020).

